# Lepromatous Leprosy Manifesting As Chronic Macrocheilia: Report of a Rare Case

**DOI:** 10.7759/cureus.47859

**Published:** 2023-10-28

**Authors:** Rajat Panigrahi, Smita R Priyadarshini, Pradyumna K Sahoo, Tanveer Alam, Shazina Saeed, Shamimul Hasan

**Affiliations:** 1 Department of Oral Medicine and Radiology, Institute of Dental Sciences, Siksha O Anusandhan University, Bhubaneswar, IND; 2 Department of Prosthodontics, Institute of Dental Sciences, Siksha O Anusandhan University, Bhubaneswar, IND; 3 Department of Dental Surgery, College of Dentistry King Khalid University, Abha, SAU; 4 Department of Epidemiology and Public Health, Amity Institute of Public Health & Hospital Administration, Amity University, Noida, IND; 5 Department of Oral Medicine and Radiology, Faculty of Dentistry, Jamia Millia Islamia, New Delhi, IND

**Keywords:** public health care, oral and maxillofacial pathology, granulomatous disorder, lepromatous leprosy, macrocheilia

## Abstract

Leprosy is a chronic debilitating disorder caused by the acid-fast bacilli *Mycobacterium leprae *(*M. leprae*) and *Mycobacterium lepromatosis*. These bacilli exhibit a distinctive predilection for the skin and peripheral nerves, although they can potentially impact any system in the body. Lately, there has been a notable reduction in mucosal symptoms, largely attributed to the timely diagnosis and treatment of leprosy. Nonetheless, oral lesions continue to hold significant epidemiological importance due to their crucial role in disease transmission. Oral manifestations, although rare, are frequently encountered in individuals afflicted with multi-bacillary leprosy. Chronic macrocheilia is an exceedingly rare manifestation of the disease, with only a few documented case reports and case studies.

This article aims to document an exceptionally uncommon case of lepromatous leprosy with chronic macrocheilia as the sole presenting feature.

## Introduction

Leprosy, a chronic infectious debilitating disorder, is caused by the acid-fast bacilli (ACB) *Mycobacterium leprae* (*M. leprae*) and *Mycobacterium lepromatosis* and primarily affects the skin and peripheral nerves [1]. Nonetheless, there exists significant clinical diversity among leprosy patients, with potential involvement of the mucous membranes, eyes, and bones [2].

Following the implementation of multidrug therapy (MDT), there was a significant reduction in the reported cases of leprosy, dropping from over five million in the 1980s to 133,802 cases in 2021, and a prevalence of 16.9 per million in the population [3].

Despite the various control measures, such as the extensive MDT use and the recent decline in the documented new cases rates, leprosy remains an endemic problem in many developing nations, with new cases emerging in nations across Southeast Asia, Africa, America, Eastern Pacific, and the Western Mediterranean. India, Brazil, and Indonesia consistently recorded the highest number of new cases, each surpassing the 10,000-case mark [2,4].

The precise mechanisms of *M. leprae* transmission are not completely elucidated, but there is evidence suggesting that the primary mode of disease transmission involves the upper respiratory tract. Prolonged close contact with an untreated patient suffering from multi-bacillary (MB) leprosy is likely necessary, potentially through infectious aerosols [5].

Rabello was the first to categorize leprosy, identifying its initial manifestation as indeterminate leprosy. Furthermore, he introduced the concept of disease polarity, elucidating that in the absence of treatment, those in the early stages of leprosy could develop either the tuberculoid (TT) or lepromatous (LL) forms of the condition [6]. In 1953, during the International Congress on Leprosy in Madrid, Rabello's classification was retained, but it was expanded to include the description of less stable forms known as "borderline" [7].

The Ridley-Jopling classification system uses the concept of spectral leprosy and categorizes leprosy into two extremes and an intermediate category, taking into account the clinical and histological features and immune status of the patient. The classification encompasses polar tuberculoid leprosy, borderline tuberculoid leprosy (BT), mid-borderline leprosy (BB), borderline lepromatous leprosy (BL), and lepromatous leprosy [4,8]. 

At the tuberculoid pole of this disease, a well-expressed cell-mediated immunity and delayed hypersensitivity effectively control bacillary multiplication through the formation of epithelioid cell granulomas. Clinically, these patients present with few (usually less than five) hypoesthetic or anesthetic skin lesions along with asymmetrical nerve involvement, and the skin biopsy shows granulomas surrounding neurovascular bundles in the papillary zone with no detectable AFB. The lepromatous form, on the other hand, has cellular anergy toward *M. leprae*, resulting in abundant bacillary multiplication and inactivated macrophages [5].

Lepromatous leprosy patients present with numerous symmetrical skin lesions along with symmetrical nerve thickening and glove and stocking anesthesia. On histopathology, the lepromatous pole presents numerous foamy macrophages, along with a few lymphocytes and plasma cells in the dermis, and numerous AFBs, singly or in clumps (globi) [2,4].

The borderline forms fall between the two aforementioned forms, occurring in patients with varying degrees of immune response. Lastly, indeterminate leprosy is considered the initial stage of the disease [2,4]. 

The clinical classification of leprosy by the World Health Organization (WHO) depends on the number of skin lesions and the involvement of nerves. Patients presenting with one to five skin lesions, no evidence of bacilli in a slit-skin smear (SSS), and enlargement of only one nerve are categorized as pauci-bacillary (PB). On the other hand, patients with six or more skin lesions or a positive SSS result (irrespective of the number of lesions) or enlargement of more than one nerve fall into the MB category [9,10,11].

Historically, the disease was characterized by distinctive facial deformities and mucosal symptoms [12]. However, recent years have witnessed a decrease in the prevalence of mucosal lesions attributed to timely diagnosis and management [13]. 

Although rare, oral manifestations of leprosy do exist [14]. However, considering the role of the upper respiratory tract in disease transmission, unraveling the oral manifestations of leprosy would enhance disease comprehension [15]. The diverse array of leprosy symptoms and their interplay with the host's immune response underscore the importance of advancements in serum- and saliva-based screening techniques in the early detection and control of the disease. Moreover, chronic oral infections can trigger the inflammatory response in leprosy, ultimately causing significant nerve damage [16].

In leprosy, the typical oral manifestation predominantly involves the hard palate infiltration, and subsequently, the soft palate, tongue, lips, gingiva, and buccal mucosa may be affected [12,17]. Mucosal lesions may exhibit diverse morphological features, including papular-nodular lesions, depapillated tongue, ulcers, gingivitis, and periodontitis [17,18].

Leprosy affecting the lips may manifest as chronic macrocheilia, the presence of flat-topped nodules, and microstomia. The swollen, rigid lips may be quite distinctive, raising cosmetic concerns [12,18]. Chronic macrocheilia may be the sole presenting manifestation in a few cases, thus complicating the etiological diagnosis. However, there is a dearth of published literature on chronic macrocheilia, primarily focusing on orofacial granulomatosis [19].

This paper aims to report an extremely rare case of lepromatous leprosy with chronic macrocheilia as the sole presenting feature. Chronic macrocheilia may be an uncommon and unusual manifestation of leprosy. Leprosy should always be considered in the differential diagnosis of asymptomatic lip swellings, particularly in endemic regions. An oral health physician must be cognizant of the varied orofacial manifestations of leprosy, thus providing a timely diagnosis and treatment.

## Case presentation

A 42-year-old male patient reported to our OPD for the evaluation of persistent enlargement of the upper and lower lips for the past 3.5 months. History revealed that the patient noticed that the enlargement was insidious in origin and progressed gradually to reach its current size. There was no discernible history of trauma, insect bites, allergies to food or drugs, or any evidence of self-induced lip or cheek biting. His personal, medical, and family history did not reveal any significant information. Additionally, there was no record of fever, cough, weight loss, or hemoptysis in the patient's medical history. The patient had sought consultation with a private healthcare provider and was given a prescription. The prescription included the use of tablets Betnesol (betamethasone, 0.5mg twice daily for 10 days), Allegra (fexofenadine, 60mg twice daily for 10 days), and Monodox (doxycycline, 100mg twice daily for 10 days). Nonetheless, the patient did not respond to the treatment, and there was no significant reduction in lip swelling.

**Figure 1:**
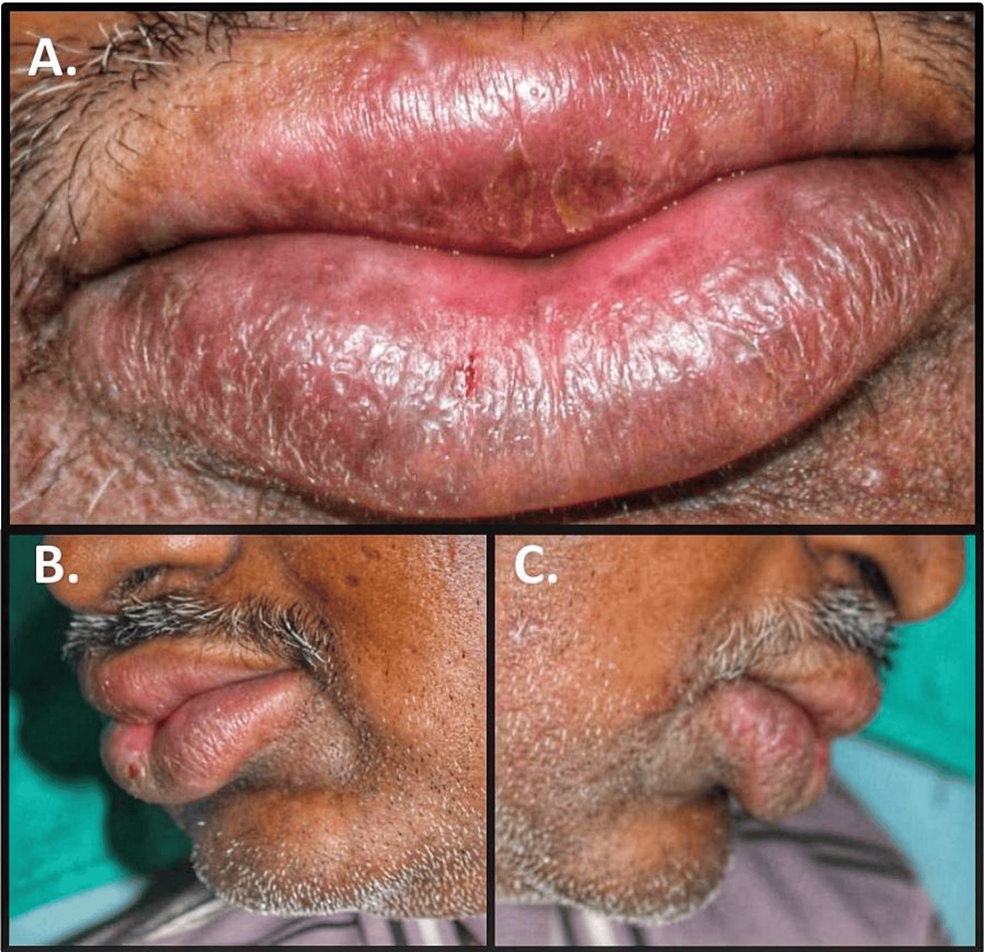
Clinical picture of the patient The clinical picture shows diffuse enlargement of the upper and lower lip with plaque-like lesions (A). The profile view shows diffuse edematous lips (B and C).

The physical examination was non-contributory, with no nodal enlargement. Extraoral examination revealed diffuse enlargement of the upper and lower lips. Lips were competent, dry, crusted, smooth, shiny, and firm. Erythematous, non-scaly, non-itchy plaque lesions with melanin pigmentation were also observed on the lips. The lower lip exhibited mild central clefting with bleeding points from cracks. There were no signs of tenderness, discharge, or altered sensations in the lip region (Figures 1A-1C).

**Figure 2:**
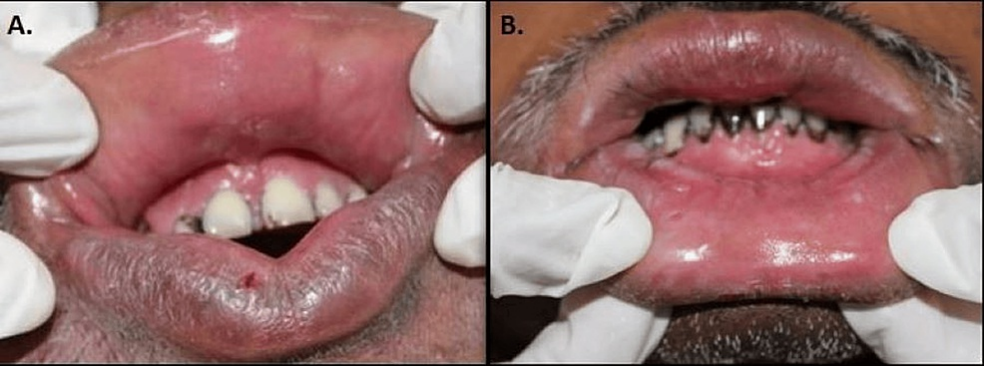
Intraoral clinical pictures Intraoral pictures show enlarged lips with areas of keratosis (A and B).

On intraoral examination, the upper and lower labial mucosa appeared enlarged and granular, with interspersed areas of keratosis. The labial mucosa was firm, non-tender, non-pinchable, and granular on digital palpation. The maxillary and mandibular anterior tooth regions showed mildly swollen labial gingiva with bleeding on probing (Figures 2A-2B).

Based on an asymptomatic, persistent lip swelling that did not improve with oral antihistamines and steroids and a non-significant medical history of food or drug allergies, fever, cough, hemoptysis, or skin diseases, along with a thorough clinical examination, a provisional diagnosis of orofacial granulomatosis was established. Tuberculosis, leprosy, leishmaniasis, Crohn's disease, and sarcoidosis were included in the list of potential differential diagnoses. The patient reported hematological, biochemical, and radiographic investigations that had been conducted a week prior, following the private practitioner's recommendation. Hematological and biochemical tests showed results within the normal range, except for a mild elevation in the erythrocyte sedimentation rate (28 mm in the first hour of Wintrobe). The Mantoux test showed a non-reactive result, and a chest radiograph (posteroanterior (PA) view) revealed normal lung fields and bronchovascular markings bilaterally. The patient was advised to undergo ultrasonography of the upper and lower lips, but the patient declined to undergo the procedure.

**Figure 3:**
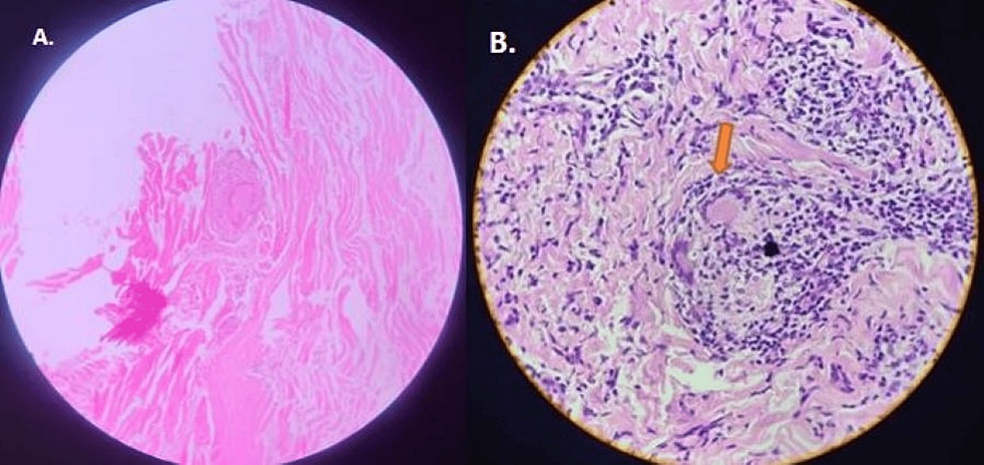
Histopathological photomicrograph Photomicrograph showing non-necrotizing epitheloid granulomas with lymphocytes and Langhans-type giant cells around the nerve bundles (arrow mark). H & E staining: A-10x, B-40x.

After informed written consent, a punch biopsy was obtained from the lesion and submitted for histopathological evaluation. The microsection revealed a normal stratified squamous epithelium. The underlying fibro-cellular dermis contained well-formed non-necrotizing epitheloid cell granulomas with foam cells, lymphocytes, and Langhans-type giant cells around the adnexa and nerve bundles (Figures 3A-3B).

**Figure 4:**
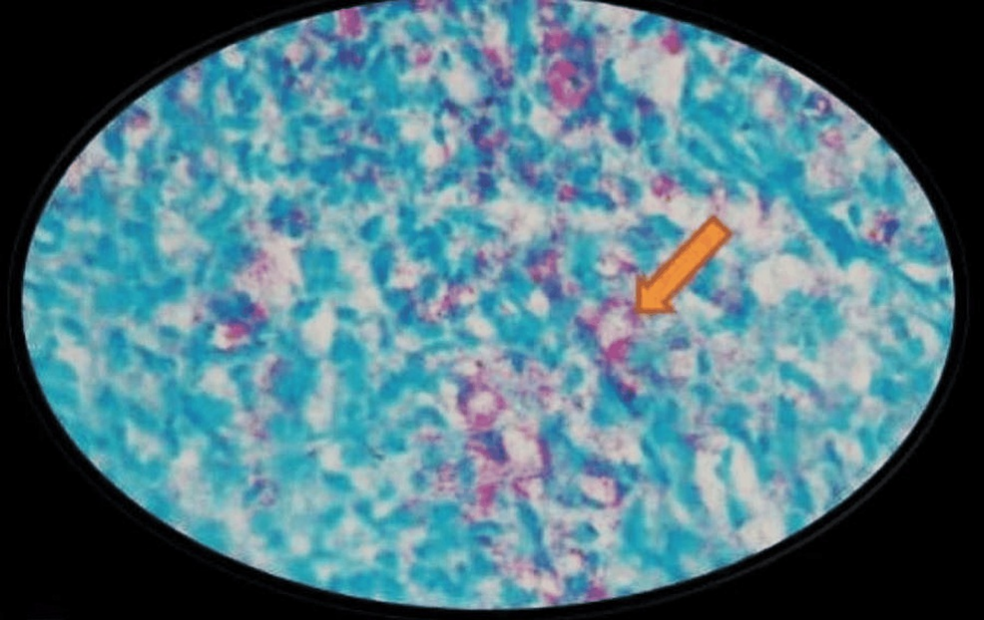
Wade-Fite staining Photomicrograph showing numerous acid-fast bacilli (pink color).

The patient was further advised for the Wade-Fite stain, which revealed numerous irregularly shaped ACB, thus establishing a confirmatory diagnosis of lepromatous leprosy (Figure 4).

**Figure 5:**
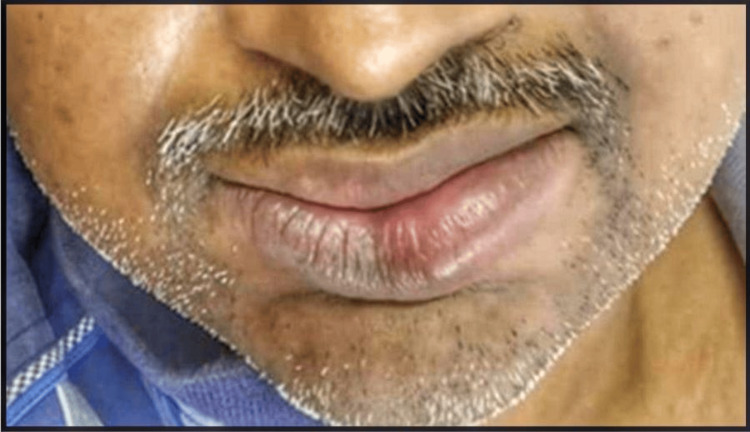
A post-treatment picture shows a marked reduction in lip enlargement.

The patient was referred to the department of internal medicine and was prescribed the World Health Organization's MB-MDT. A follow-up after three months revealed a notable decrease in lip swelling (Figure 5).

The MDT was further extended for 12 months, and the patient is presently receiving tele-consultations.

## Discussion

A detailed literature search carried out on the Google Scholar and PubMed search engines revealed only seven case reports [17,20-25], and two retrospective studies [19,26] of chronic macrocheilia as the manifestation of leprosy (Table 1).

**Table 1:** Case reports and case studies on leprosy manifesting as chronic macrocheilia MB-MDT: multibacillary-multi drug therapy; WHO: World Health Organization; pred: prednisolone; CG: cheilitis granulomatosa; M: male; F: female

No.	Author(s) and year	Case report/study	Age (years)/sex	Presenting manifestation	Other associated features	Diagnosis and treatment
	Raghavendra Rao et al., 2013 [20]	Case report	53/M	Asymptomatic upper lip swelling of four-month duration	Bilateral infiltrating plaques at the angles of the mouth	Borderline tuberculoid leprosy; treated with WHO MB-MDT and oral pred.
	Gogri AA et al., 2015 [21]	Case report	17/M	Asymptomatic upper and lower lip swelling of seven-month duration	Hypopigmentation at the center of the upper lip; inflamed and swollen labial gingiva; bilateral scaling and erythema on the cheeks; nerve thickening and hyperesthesia in the chin area	Initially diagnosed as CG, the patient was treated with systemic and intralesional steroids. Later, it was diagnosed as borderline lepromatous leprosy with a type I reaction and treated with MB-MDT.
3.	Lunge SB et al., 2021 [22]	Case report	25/M	Asymptomatic swelling of the upper and lower lips of seven-month duration with a waxing and waning pattern	Erythema over the cheeks	Borderline tuberculoid leprosy recalcitrant to MB-MDT treated with second-line drugs ofloxacin, minocycline, clarithromycin, and oral pred.
4.	Sadasivamohan A, et al. 2022 [23]	Case report	40/F	Ill-defined, edematous, soft, non-tender plaque over the right upper lip.	NA	Borderline tuberculoid leprosy; treated with MB-MDT
5.	Situala S et al. 2022 [24]	Case report	50/F	Persistent swelling of the lower lip	Erythematous, non-pruritic, non-scaling plaque over the upper lip and chin	Initially diagnosed as CG, and treated with intralesional steroids. Later, it was diagnosed as borderline tuberculoid leprosy and treated with MB-MDT.
6.	Gupta I et al., 2023 [25]	Case report	70/F	Swollen lower lip and an elevated erythematous chin lesion.	Erythematous, infiltrated plaque on the lower lip extending up to the angle of the mouth.	Borderline tuberculoid leprosy, treated with MB-MDT and oral steroids
7.	Yadav S et al., 2023 [17]	Case report	21/M	Asymptomatic swelling of the lower lip with fissuring, inflamed and swollen labial gingiva	Well-defined, hyperesthetic, hypopigmented plaques over the chin and cheeks.	Borderline tuberculoid leprosy and treated with MB-MDT.
8.	Handa S et al., 2002 [26]	Retrospective study	Three patients (two males & one female) Mean age: 41.3 ± 24.2 years	Two patients with leprosy presented with sudden onset ‘waxing and waning’ lip swelling.	Mild local tenderness, warmth, and scaling of the lesions.	Borderline tuberculoid leprosy with type 1 lepra reaction
				Slowly progressive labial enlargement	Bilateral facial nerve paralysis	Borderline lepromatous leprosy
9.	Toumi A et al., 2021 [19]	Retrospective study	78/M		Infiltrated erythematous plaques, firm erythematous nodules, and hypoesthesia of the extremities	Lepromatous leprosy

Oral lesions in leprosy are usually asymptomatic, tend to progress gradually, and may occur due to the hematogenous or lymphatic spread of *M. leprae* or as a secondary consequence of nasal lesions [16,18,27]. 

The prevalence of oral leprotic lesions is a subject of considerable dispute in the literature, with reported rates varying from 0% to 70% of cases [2,13,18,28,29]. Oral lesions are more frequently seen in multibacillary lepromatous leprosy, and they typically present as a late-stage manifestation of advanced leprosy [2,4,9,12,18,28]. However. Ghosh et al. described orofacial lesions in the borderline-tuberculoid form [30]. Published literature has documented a range of oro-facial lesions of leprosy [12,16,18,30] (Table 2).

**Table 2:** Orofacial manifestations of leprosy

S.no.	Affected organ	Presenting features
1.	Skin and cutaneous adnexa [16,30]	Macular/raised, well-defined, hypopigmented, anhidrotic, and paresthetic lesions; Infiltrating lesions form plaques and nodules (lepromas). Advanced stages present as “facies leonine” with loss of eyebrows and eyelashes (madarosis). Edema of the face and extremities
2.	Nervous system [16,30]	Peripheral neuropathy: thickened, irregular, and painful nerves, hypoesthesia or anesthesia, decreased muscle strength, amyotrophy, paralysis of facial and trigeminal nerves, usually maxillary division claw, hand, and wrist drop (due to nerve lesions of the upper limb), neuritis often causes sequelae and may lead to chronic pain along the affected nerves, which is called neuropathic pain. Involvement of the buccal and mandibular branches of the facial nerve may cause mastication and speech implications.
3.	Face, nose, and ears [12,16,30]	Destruction of facial cartilage or bones, primarily the naso-maxillary complex. Facies leprosa (facial nodules, inflammatory endonasal changes, atrophy of the alveolar pre-maxillary processes and nasal spine, with loss of maxillary incisors). A saddle-shaped nose if the nasal septum is destroyed. Thickening of ear lobes, thinned and sagged ears, ear deformities, and joint stiffness
4.	Oral cavity [12,18,30]	Leprosy non-specific lesions: exanthem of the palate or uvula, erythematous candidiasis, fissured tongue, and inflammatory fibrous hyperplasia
		Leprosy-specific lesions: hard and soft palate loss, shininess of the mucosa, erythematous papules, nodular sub-mucosal infiltrate, ulceration, and perforation of the palate. Tongue: multiple superficial ulcers; mild glossitis; atrophy or loss of papillae with loss of taste; fissured tongue; nodules on the anterior tongue (pavement-like appearance). Buccal mucosa diffuse infiltration, papulo-nodules lesions, ulceration lips flat-topped nodules, macrocheilia, microstomia gingiva gingival bleeding, and papillary hypertrophy of the gums uvula and faucial pillars fibrosis with the partial or complete destruction of the uvula, miliary papules and nodules, triangular deformity of fauces
5.	Teeth and supporting structures [12,18,30]	Described as odontodysplasia leprosa; early and severe granulomatous involvement of the pre-maxilla in childhood; circumferential hypoplasia is the shortening of roots, usually involving maxillary anterior teeth; long-standing lepromatous lesions may show granulomatous invasion of the pulp and pinkish discoloration of the crowns. Dental caries, gingivitis, periodontitis, periodontoclasia, and periapical granulomas

*Mycobacterium leprae* tends to multiply in cooler regions with an estimated mean surface temperature of 27.4°C. The reduction in intraoral temperature is primarily attributed to the initial nasal invasion by the bacilli, resulting in nasal blockage and prompting individuals to engage in mouth breathing, thus permitting the inspired air to reduce the temperature [12,18,27].

Macrocheilia is an inflammatory disorder associated with the enlargement of either one or both lips, causing persistent swelling that may lead to cosmetic disfigurement. Additionally, it frequently interferes with daily functions such as phonetics and mastication [19]. Nearly all the patients exhibit a gradual increase in the size of one or both lips, and the absence of additional features in a majority of cases often poses a diagnostic threat to the oral health practitioner [26].

Chronic macrocheilia (CM), defined as lasting for at least eight weeks, can have diverse causes, one of which is orofacial granulomatosis (OFG). Orofacial granulomatosis is an uncommon, chronic disorder characterized by the presence of orofacial granulomas. Primary or idiopathic OFG encompasses cheilitis granulomatosa of Miescher (CGM) and Melkersson-Rosenthal syndrome (MRS). Secondary OFG may be associated with infectious diseases (such as leishmaniasis, tuberculosis (TB), leprosy, and deep fungal infections), Crohn's disease, and sarcoidosis. Iatrogenic CM can be associated with foreign body responses to cosmetic fillers. Other reported causes of CM may encompass benign and malignant tumors (minor salivary gland tumors, hemangiomas, lymphangiomas, and lymphomas), amyloidosis, and glandular cheilitis [19,26]. Wiesenfeld (1985) pioneered the concept of OFG to include disorders causing granulomas in the orofacial region without any accompanying systemic symptoms [21,31]. Cheilitis granulomatosa, initially described by Miescher, is an inflammatory disorder with an obscure etiology. It manifests as a mono-symptomatic variant of MRS, characterized by chronic localized swelling of one or both lips. The simultaneous occurrence of CGM with a fissured tongue and relapsing facial palsy characterizes the Melkerson-Rosenthal syndrome [25,26]. 

Chronic macrocheilia resulting from leprosy closely simulates MRS since both conditions clinically exhibit CGM, facial palsy, and granulomas in histological examinations [25]. Facial paralysis can manifest in both MRS and leprosy, as trigeminal nerve paralysis has been documented in leprosy cases. However, the presence of a distinctively fissured tongue is a diagnostic hallmark of MRS [21,24].

Both leprosy and CGM exhibit waxing and waning patterns of lip enlargement. In leprosy, the accompanying skin lesions exhibit warmth, tenderness, and scaliness. Moreover, the lesion's margin extends beyond the lip, providing subtle distinctions that differentiate it from CGM [24,26]. Microscopically, both leprosy and CGM exhibit non-caseating granulomas with multinucleated Langhans-type giant cells. Furthermore, CGM is distinguished by perivascular mononuclear infiltration, lymphedema, and fibrosis. On the contrary, leprosy is distinguished by upper dermis granulomas and perineural and periadenexal infiltrates consisting of foamy cells, plasma cells, and lymphocytes [24,32]. In the case presented, MRS may be ruled out by the absence of facial paralysis and a fissured tongue. The lip lesions were dry and scaly and exhibited warmth on palpation, thus possibly negating the diagnosis of CGM.

Leishmaniasis is usually seen in young individuals in endemic regions and presents as partial lip swelling with central crusted ulcerations [19]. However, our patient presented with diffuse enlargement of the upper and lower lips.

Crohn’s disease (CD) should be included in the differential diagnosis when macrocheilia is seen in young patients (<16 years), accompanied by oral ulcers and elevated inflammatory markers [19]. Chron's disease is characterized by crampy suprapubic abdominal pain, non-bloody diarrhea with refractory fever, appetite and weight loss, and malaise. Salient oral features include chronic lip swellings (macrocheilia), cobblestoning of the oral mucosa, deep oral ulcers, mucogingivitis, and tissue tags. A segmented and intermittently distributed pattern of abdominal lesions during the endoscopic evaluation is diagnostic for CD [33]. Crohn's disease could be ruled out as the patient did not present with gastrointestinal symptoms or signs of malabsorption or anemia.

Tuberculosis, a chronic granulomatous infectious disorder caused by *Mycobacterium tuberculosis*, is mainly contracted through the inhalation of *Mycobacterium*-laden airborne droplets [34]. Oral TB is typically characterized by the presence of ulcers, while macrocheilia is seen as a relatively rare phenomenon. Tuberculosis patients typically present at a younger age and commonly show signs of regional lymphadenopathy. Additionally, a higher number of lymph nodes are affected in TB, and these nodes tend to be larger [26]. Caseating granulomas encircled by epithelial cells, lymphocytes, and multinucleated giant cells establish the histological diagnosis [35]. A negative history of fever, chronic cough, weight loss, and anorexia, with a non-reactive Montoux test and normal chest radiograph, may possibly rule out TB.

Sarcoidosis is a systemic granulomatous condition that can involve multiple organs, with a distinctive predilection for the lungs and intrathoracic lymph nodes [36]. Orofacial lesions, although uncommon, may manifest as persistent dry mouth and enlargement of major salivary glands and gingiva. Chronic macrocheilia in sarcoidosis is rarely seen and described as a gradual and progressive increase in lip size rather than sudden edematous episodes [37]. Sarcoidosis is diagnosed based on the cardinal clinical manifestations, histological presence of non-caseating granulomas, and exclusion of other causes of granulomatous inflammation [36]. Lack of regional lymphadenopathy, biochemical investigations within the normal range, angiotensin-converting enzyme levels (ACE), and a normal chest radiograph negated the likelihood of sarcoidosis.

Angioedema is typically characterized by non-pitting, asymmetric swelling of the face, lips, tongue, larynx, genitalia, and extremities. Angioedema typically occurs within several minutes of exposure to an identifiable allergic trigger, including food, medications, and insect stings. It rapidly subsides after administration of antihistamines and epinephrine and does not recur without repeat insult by the allergic trigger [38]. Angioedema could be negated as the patient presented with an asymptomatic, persistent lip enlargement, was non-responsive to systemic antihistamines and steroids, and had no identifiable trigger factors.

Hemangioma could be negated based on the patient's age and gender (hemangiomas typically appear in infancy and young adults, especially in females), lack of a distinctively bright red appearance, and a negative diascopy test [39].

Lymphangioma, a benign hamartomatous lesion of the lymphatic system, primarily affects infants and children and involves the tongue, although a few lip cases have also been documented. The lip lesions frequently exhibit an asymmetric, asymptomatic, firm, and nodular pattern [38].

**Figure 6:**
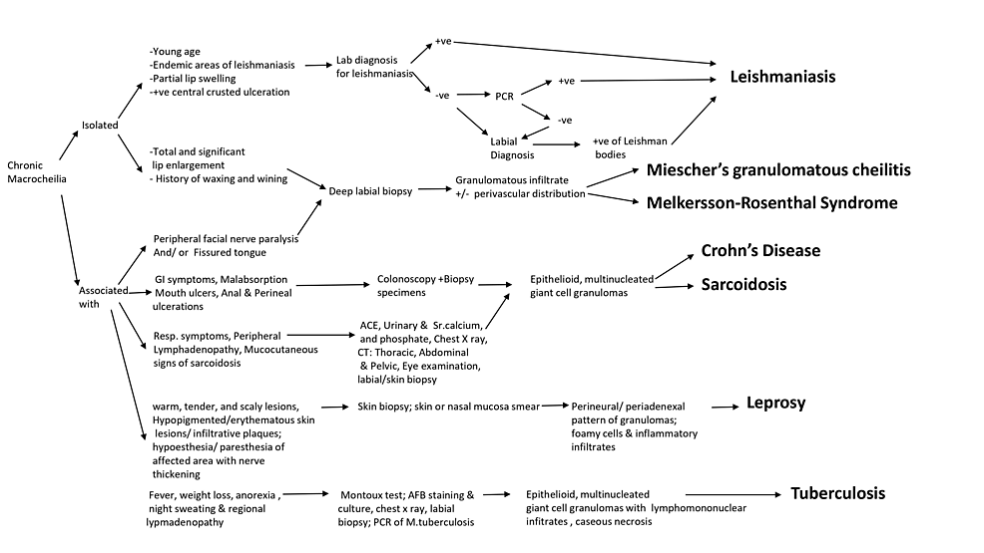
Etiological diagnostic algorithm for chronic macrocheilia Source: The figure has been created by the authors of this article using the information from Taumi et al. [19]. AFB: acid-fast bacilli; ACE: angiotensin-converting enzyme; GI: gastrointestinal; CT: computed tomography; PCR: polymerase chain reaction

A descriptive etiological diagnostic algorithm for chronic macrocheilia is shown in Figure 6 [19].

Recommendations

Chronic macrocheilia may be a rare and atypical manifestation of leprosy. Leprosy should always be included in the differential diagnosis of asymptomatic lip swellings, particularly in endemic nations. Oral healthcare professionals should have a comprehensive understanding of the diverse orofacial manifestations of leprosy to facilitate timely diagnosis and treatment.

## Conclusions

Leprosy is a chronic infectious disorder affecting multiple organs with a distinctive predilection for the skin and peripheral nerves. Chronic macrocheilia as the sole presenting feature of leprosy is an extremely rare occurrence. Our patient reported a concern regarding chronic, progressive, asymptomatic enlargement of the lips. After a detailed anamnesis and clinicopathological evaluation, a diagnosis of chronic macrocheilia secondary to borderline tuberculoid leprosy was arrived at. The patient was prescribed the World Health Organization-recommended MB-MDT for a year. The lip swelling has considerably reduced after a three-month follow-up.
